# Insect-specific virus evolution and potential effects on vector competence

**DOI:** 10.1007/s11262-018-01629-9

**Published:** 2019-01-10

**Authors:** Pontus Öhlund, Hanna Lundén, Anne-Lie Blomström

**Affiliations:** Section of Virology, Department of Biomedical Sciences and Veterinary Public Health, Swedish University of Agricultural Sciences, Box 7028, 750 07 Uppsala, Sweden

**Keywords:** Insect-specific virus, Arbovirus, Vector competence, Evolution

## Abstract

The advancement in high-throughput sequencing technology and bioinformatics tools has spurred a new age of viral discovery. Arthropods is the largest group of animals and has shown to be a major reservoir of different viruses, including a group known as insect-specific viruses (ISVs). The majority of known ISVs have been isolated from mosquitoes and shown to belong to viral families associated with animal arbovirus pathogens, such as *Flaviviridae, Togaviridae* and *Phenuiviridae*. These insect-specific viruses have a strict tropism and are unable to replicate in vertebrate cells, these properties are interesting for many reasons. One is that these viruses could potentially be utilised as biocontrol agents using a similar strategy as for *Wolbachia*. Mosquitoes infected with the viral agent could have inferior vectorial capacity of arboviruses resulting in a decrease of circulating arboviruses of public health importance. Moreover, insect-specific viruses are thought to be ancestral to arboviruses and could be used to study the evolution of the switch from single-host to dual-host. In this review, we discuss new discoveries and hypothesis in the field of arboviruses and insect-specific viruses.

## Introduction

**Fig. 1:**
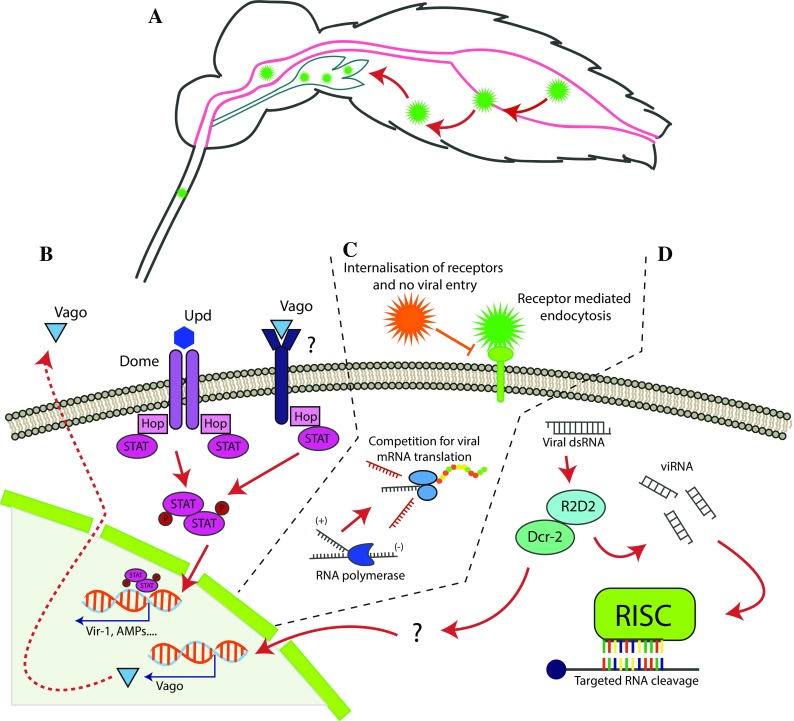
Schematic overview of some of the mosquito antiviral mechanisms. **a** The mosquito ingests an arbovirus-infectious blood meal into the midgut. The virus enters and replicate in the midgut epithelial cells, after successful replication the virus escape into the haemolymph and spread systemically including to the salivary glands, where the virus enters and replicate before being transmitted via the saliva. **b** The JAK-STAT pathway is mainly activated when the transmembrane receptor Domeless (Dome) recognise extracellular unpaired ligands (Upd) leading to a conformational change that start autophosphorylation of Hop, which in turn phosphorylates Dome. This is leads to the phosphorylation and dimerization of STAT, resulting in a translocation of STAT dimers to the nucleus which activates the transcription of specific antiviral genes. **c** A primary viral infection can block a secondary infection of a similar virus via mechanisms hypothesised to involve competition for, or modification of cellular resources reducing receptor binding, viral entry, RNA replication and translation of the secondary virus. **d** Viral dsRNA, either as replication intermediates or as part of the viral genomes, are processed by the Dcr-2-R2D2 complex to generate siRNAs of approximately 21–23 bp of length. The siRNA are incorporated into the RNA-induced silencing complex (RISC) to recognize viral RNA for degradation. dsRNA can be sensed by the Dicer-2 DEcD/H-box helicase domain and via an unknown pathway activate expression and secretion of Vago, which can activate the JAK-STAT pathway via an unknown receptor in nearby cells

The first insect-specific virus (ISV) was discovered over 40 years ago by Stollar and Thomas [1]. It was isolated from an *Aedes aegypti* cell culture where a large number of syncytia were observed and the virus was named cell fusing agent virus (CFAV). Further, when inoculated on different vertebrate cell lines no cytopathic effect (CPE) could be observed and the virus could not be re-isolated, suggesting that the virus must be insect-specific [1]. Years after its discovery CFAV was molecularly characterised as a positive-sense RNA virus within the family *Flaviviridae* [2] and in 2006 it was isolated from field-caught mosquitoes in Puerto Rico [3]. Since the CFAV discovery a large number of ISVs have been discovered with increased frequency in the last two decades due to the advancement in high-throughput sequencing, metagenomics and intensified mosquito surveillance [4]. Even though this group of viruses is called “insect-specific viruses” the majority have been discovered in mosquitoes and one can argue that for these viruses the term “mosquito-specific viruses” would be more appropriate. ISVs are restricted to arthropods and are unable to replicate in vertebrate cells [5]. Because of the host-restriction there is no vertebrate amplifying host that can maintain a successful viral lifecycle between the mosquito and vertebrate animal, which is the case for most arboviruses [6]. Therefore, the main mechanism of transmission and maintenance of ISVs is thought to be vertical transmission, in which the virus is passed transovarially from infected female mosquitoes to their offspring. This is supported by both laboratory and field studies, where offspring from ISV-infected female mosquitoes have tested positive for the correlating virus [3, 7–11]. The mechanisms for how ISVs manage to establish an infection in the mosquito is, however, not known. In the case of arbovirus interaction and infection in the mosquito more research has been done. For an arbovirus to successfully transmit to a new host via a blood meal, it needs to enter and replicate in the salivary gland of the mosquito. However, there are barriers and tissue-specific antiviral mechanisms that the virus has to overcome for this to occur (Fig. 1). The first barrier is the midgut epithelial cells. The virus needs to enter and escape these cells before spreading to the haemolymph from where it can spread systemically to the rest of the body including the salivary glands via the haemolymph circulation [12–14]. Each of these barriers have tissue specific antiviral immune responses including the Toll, immune deficiency factor (Imd), Janus kinase (JAK)- signalling transduction and activation of transcripts (STAT) pathways as well as RNA interference (RNAi) [15]. These pathways allow the mosquito to mount a defence against invading microorganisms, including viruses, and to understanding these antiviral mechanisms and control of viral infection in specific tissues is crucial and the backbone of novel control strategies that target arbovirus transmission in nature.

Of all arboviruses that cause human disease over 90% are vectored by mosquitoes [16] and with increasing temperatures, urbanization and global trade, the geographic range of different mosquitoes have expanded with associated increase in arbovirus disease burden [17–21]. Arboviruses are a global problem with, for example, annual outbreaks of dengue virus (DENV) in the Americas [22], yellow fever virus outbreaks in south America [23], West Nile virus (WNV) becoming endemic in Europe [24] and the emerging Zika virus (ZIKV) [25]. The global arbovirus disease burden and the lack of licensed drugs and vaccines, cause an urgent need of new tools for disease control. In recent years, researchers have begun to study vector competence, as evidence has suggested that the microbiome of the mosquito can alter the susceptibility of certain arboviruses. The most studied example is the endosymbiotic bacterium *Wolbachia*, which naturally infect a broad range of arthropods including many mosquito species and is maternally transmitted. *Wolbachia* has been proven to reduce the vector competence of important arboviruses in key mosquito species through various mechanisms [26]. In a similar manner, it is believed that ISVs could also have the potential to be utilised as biocontrol agents with proposing effects of superinfection exclusion, upregulation of the vector antiviral immune-response and maintenance in nature by transovarial transmission. In this review, we discuss research strengthening the hypothesis of ISVs being ancestral to arboviruses as well as provide a comprehensive description of ISVs effect on vector competence.

## Host-range restrictions of ISVs

Insect-specific viruses are, as mentioned in the introduction, characterized by their incapacity to infect vertebrates. This can be assessed through, for example, viral inoculation of mammalian, avian, or amphibian cell lines [27–29] or by intracerebral infection of neonatal mice [29–31]. Host-range restrictions may exist at both the pre- and post-entry steps of the viral cycle. To be able to replicate the virus must enter the cell, deliver their genome, replicate and build new infectious virus particles. Each of these steps is an obstacle to be overcome by the virus [32]. To study the mechanisms behind host-range restrictions reverse genetic systems have proven to be a valuable tool through the ability of creating chimeric viruses [28, 31, 33].

Insect-specific viruses are believed to be restricted by the innate immune system in the vertebrate cell, but there is evidence pointing to that this is not the only mechanism causing the restriction of host-range [34]. In contrast to arboviruses, who can replicate at temperatures up to 42 °C insect-specific viruses replicate only at ambient temperatures. In a study by Marklewitz et al. [35] replication of an insect-specific bunyavirus was decreased and even hindered by elevated temperatures in invertebrate cells where arboviral bunyaviruses were not. However, it is not sufficient to just lower the temperature to enable the replication of ISVs in mammalian cells [5, 29, 35].

The impact of the vertebrate innate immune system in host-restriction has been shown for the ISV Kamiti River virus (KRV). Knockdown of pattern recognition receptors (RIG-I, MDA5 and TLR3) led to an elevation of KRV vRNA levels in both Vero and human lung carcinoma cells (A549) which points to that several of these receptors are important in the detection and control of replication of KRV in vertebrate cells [34]. The study also showed that KRV was able to enter IRF3,5,7−/− mouse embryonic fibroblasts, translate and shed infectious particle, although at low levels, which suggests that the KRV is capable of replicating in vertebrate cells if the innate immunity pathways are silenced [34]. This is in contrast to the insect-specific alphavirus, Eilat virus (EILV), that is restricted at multiple levels. In a study from 2012 Nasar et al. showed that EILV is unable to replicate in vertebrate cells, even after genomic RNA was electroporated directly in the cytoplasm. It was hypothesized that this was due to incorrect interactions between the EILV RNA or its gene products with vertebrate cell cofactors. A study from 2015, using Sindbis virus and EILV chimeras, showed that EILV is actually blocked at two independent stages of the replication cycle, both at RNA replication step as well as at viral entry [5].

A study by Junglen et al. [33] also showed that the host-range restriction can be at several levels of the viral life cycle. In this study chimera Yellow fever virus carrying envelope proteins from the insect-specific flavivirus Niénokoué virus (NIEV) was use to study host range restrictions. In the study, they observed that the first barrier against infection of vertebrate cells was viral entry. The YF/NIEV chimera grew, and produced new virus particles in C6/36 cells after infection, however, no virus was shown after infection of BHK cells, indication that NIEV was unable to mediate viral entry into vertebrate cells. Electroporation of RNA from the YF/NIEV clone showed growth and assembly of infectious particles in C6/36 but no replication nor assembly was seen in BHK cells indicating that NIEV is restricted at both attachment/entry as well as at the assembly /release level [33].

## Insect-specific virus evolution

ISVs and arboviruses both infect and replicate in insect vectors and show evolutionary relationship. This fact suggests that arboviruses could have been ISVs that through evolution acquired the ability to expand their host-range to also include vertebrates. As mentioned in the introduction, almost all arboviruses and insect-specific viruses belong to the RNA virus order *Bunyavirales* (negative (−) sense ssRNA), or to the viral families *Flaviviridae* (postitive (+) sense, ssRNA), *Reoviridae* (dsRNA), *Rhabodoviridae* (−ssRNA) and *Togaviridae* (+ssRNA). RNA viruses lack the same proofreading mechanism as DNA viruses which results in greater plasticity and higher mutation rates [36]. Together with insect’s affinity to live in large dense populations this could be one of the explanations to the great diversity of arbovirus hosts. Adaptations that provide a virus with the possibility to infect new hosts gives the virus the possibility to spread geographically. Altogether, this suggests that ISVs are a potential source of new arboviruses.

The idea that arboviruses originated from viruses found in arthropods is far from new, as it was first suggested more than 50 years ago [4]. Abundance of viral RNA in the arthropod transcriptome and high incidence of endogenous copies in the genome of arthropods suggests that ISVs have probably played a role in the evolution of RNA viruses [37]. Together with the fact that many insect-specific viruses appear to be vertically transmitted, this points to the fact that these viruses coexisted with their insect host for a long period of time [35, 38–41]. Reconstruction of phylogenetic ancestral hosts helps in the identification of ancestral host switching processes. It can also help predict in which direction the virus will spread to new hosts [42].

### Bunyavirales

Arthropod hosts have been constructed for all deep tree nodes of the bunyavirus tree [35], which suggest that arboviruses from this order evolved from insect-specific viruses. It has also been suggested that an ancient insect-borne bunyavirus lineage made the jump from insects to mammals and that these viruses comprise the present-day genus *Hantavirus* [43]. Ballinger et al. [38] shows evidence for that the insect-specific phasmaviruses are members of an ancient bunyavirus lineage grouping with Hantavirus, Orthobunyavirus, and Tospovirus. Bunyaviruses is a large and diverse genus composed of more than 350 viruses. Many of the bunyaviruses are pathogenic to human and animals but the genus also comprises several insect-specific viruses. These viruses are often divergent from arthropod viruses. Recent studies have discovered a number of insect-specific bunyaviruses, and furthermore some studies have also found nucleoproteins similar to bunyaviruses in the genome of non-blood feeding insects such as *Drosophila*. This finding suggests that there have been interactions between bunyaviruses and arthropods for over 20 million years [27, 37, 38, 44–48]. In a study by Marklewitz et al. [35] a reconstruction of an ancestral host for *Bunyavirales* was made. The results in the study points to that vertebrate, or dual-host tropism, have evolved several times during the evolution of viruses. For the *Hantavirus*, spread primarily by rodents, arthropod tropism is thought to have been lost in favour of vertebrate single tropism [35].

### Flaviviridae

One of the first discovered insect-specific clades of flaviviruses, that includes cell fusing agent virus, Kamiti River virus and Culex flavivirus, branches at the base of the Flavivirus genus suggesting an arthropod virus origin of the arboviruses within the genus [4, 49, 50]. The same could be suggested for all flaviviruses as 12 flavivirus-like viruses of deep rooting lineages were identified in a range of arthropod species [51]. A second clade of insect-specific flaviviruses that cluster with the mosquito-borne flaviviruses have been found, although it is unclear at this point whether they lost dual-host tropism or maintained single host tropism from the root of the genus [4, 29, 50, 52].

### Togaviridae

Two insect-specific viruses identified within the Alphavirus genus (*Togaviridae*): Eilat virus and Taï Forest alphavirus are both related basally to Western equine encephalitis virus which could suggest an insect-specific ancestor to Western equine encephalitis virus [28, 53]. Yet, the rarity of insect-specific alphaviruses makes conclusions concerning the evolution in this genus hard to draw.

### Rhabdoviridae

There are several insect-specific lineages in a number of places in the *Rhabdoviridae* family phylogenetic tree [54]. However, the existence of the bat- and human pathogenic lyssaviruses and the novirhabdoviruses, known to infect aquatic hosts, that are both situated in basal phylogenetic positions, could coincide with the idea of an insect-specific ancestor in the *Rhabdoviridae* family [32].

Insights in the cellular and genetic requirements for adaptation of insect-specific viruses to vertebrate hosts are of great importance. This information can potentially be used to predict potential future spill over of new pathogenic viruses from insects to humans and animals. Furthermore, there is great value in the knowledge of the origin and evolution of viruses, as it provides insight into the history of virus emergence and how viruses acquire the ability to infect new hosts and become more pathogenic.

## Vector competence studies

Today mosquitoes are the subject of a wide variety of control strategies utilizing everything from bacteria, aquatic animals and chemicals [55–57]. Most of these strategies focus on reducing the vector abundance, often by targeting and killing mosquito larvae and/or adults. These strategies reduce or contain arbovirus transmission, but are cumbersome and costly. Mainly because of the dependence on community participation to access hidden breeding sites such as yards or gardens and the constant need to repeat the treatments and monitor levels of larvae and/or adults [58, 59]. To measure how effectively a hematophagous arthropod vector can transmit a virus (vectorial capacity) we need to consider several factors such as vector competence (the proportion of vectors that acquire an arbovirus infection and transmit it to a vertebrate), vector abundance, host specificity, vector longevity, the extrinsic incubation period and blood feeding frequency. All these factors have an impact on vectorial capacity and arbovirus disease burden and can be calculated with the equation

$$C=\left( {{\text{m}}{{\text{a}}^2}} \right)\left( {{\text{pn}}} \right)\left( b \right)/ - \log \left( p \right),$$

where *C* is the vectorial capacity, *b* is the vector competence, *p* is the daily probability of vector survival, *n* is the extrinsic incubation period and ma^2^ is a combined value of blood feeding frequency with human biting rate [60, 61].

In recent years, researchers have looked at other options such as genetically modified vectors (GMVs) [62] and manipulation of the microbiome to enhance the antiviral resistance [63]. These strategies are focusing on the vector competence of the mosquito to carry and transmit arboviruses of human and animal importance. One advantage of these novel control strategies is the maintenance in the mosquito population, were both the GMVs and manipulation of the microbiome are vertically transmitted by female mosquitoes to their offspring [7, 64, 65] and thus sustaining the transmission blocking effect. Manipulation of the microbiome can be done by altering the flora of bacteria, fungi or virus in the mosquito by, for example, introducing a microorganism with a known interfering effect. The most studied example is the use of the endosymbiotic bacterium named *Wolbachia*, which has repeatedly proven the ability to hinder infection of important arboviruses such as chikungunya virus (CHIKV), DENV, ZIKV and WNV [26, 66–68]. Moreover, the microbiome of the mosquito and specifically the midgut microbiome has shown to contribute to antiviral protection through several mechanisms. It serves as a physical barrier by blocking gut epithelial cells from pathogenic exposure [69], the microorganisms can produce secondary metabolites that inhibit arboviruses [70] and it also provides a basal immune activation, aggravating viral entry and replication [71, 72]. In this review, we will focus on the potential use of ISVs as biocontrol agents (Table 1) in a similar manner as *Wolbachia*. The relative genetic similarity between ISVs and arboviruses have a potential for replicative interference not only through upregulation of antiviral immune-responses in the vector but also via a phenomenon called superinfection exclusion (Fig. 1c). Superinfection exclusion (or homologous interference) is a phenomenon where a primary viral infection can block a secondary infection of a similar virus. The molecular mechanisms of superinfection exclusion are hypothesised to involve competition for, or modification of cellular resources reducing receptor binding, viral entry, RNA replication and translation of the secondary virus [73]. Superinfection exclusion has, for example, been demonstrated for two different strain of WNV in *Culex pipiens* mosquitoes [74], WNV and St. Louis encephalitis virus (SLEV) in *Culex quinquefasciatus* [75] and in many different cell culture superinfection experiments [76–78].

**Table 1:** Summary of the papers discussed in the “Vector competence studies” subsection regarding the effect of different ISVs on vector competence

Study	ISV	In vitro	In vivo	Control virus	Effect	Year	References
Kent et al.	CxFV	Yes	Yes	WNV	No effect on vector competence for WNV	2010	[89]
Bolling et al.	CxFV	Yes	Yes	WNV	Early interference of infection, no effect on transmission	2012	[65]
Hopson-Peter et al.	PCV	Yes	No	WNV, MVEV	10–43-fold growth inhibition in C6/36 cells	2013	[97]
Kenney et al.	NHUV	Yes	No	WNV, SLEV, JEV	1.5 million-fold reduction for WNV, 80-fold reduction for JEV and 15,000-fold reduction for SLEV in C6/36	2014	[99]
Goenaga et al.	NHUV	Yes	Yes	WNV	4000-fold reduction for WNV in vitro. No significant effect in co-infected *Cx. pipiens. However, Co*-infected *Cx. quinquefaciatus* had a significant difference in transmission at 7 and 9 dpi	2015	[101]
Kuwata et al.	CxFV	Yes	No	JEV, DENV	Pre-existing CxFV infection do not suppress growth of a superinfecting flavivirus but rather enhance virus release from the NIID-CTR cells	2015	[93]
Nasar et al.	EILV	Yes	Yes	SINV, VEEV, EEEV, WEEV, CHIKV	In vitro results showed that EILV infection reduced superinfecting virus production by 10 to 10,000-fold and delayed replication kinetics at least 12–48 h regardless of virus or MOI. In vivo studies showed a delay of dissemination from the midgut by 3 days	2015	[31]
Hall-Mendelin et al.	PCV	No	Yes	WNV	Significantly lower infection rate in PCV-infected *Cx. Annulirostris* orally exposed to WNV	2016	[98]
Talavera et al.	CxFV	No	Yes	RFV	No effect	2018	[94]
Schultz et al.	CFAV, PCLV	Yes	No	ZIKV, DENV & LACV	90% reduction of ZIKV & DENV growth. Complete inhibition of LACV at MOI 0.1	2018	[104]

### Culex flavivirus

The ISV Culex flavivirus (CxFV) was first isolated in Japan in 2007 [79] and has since been isolated in *Culex* mosquitoes all over the globe [80–88]. Kent et al. was the first to evaluate the potential effect of CxFV to block propagation and transmission of arbovirus. In that study, prior infection of CxFV Izabal was evaluated for its effect on vector competence of WNV in *Culex quinquefasciatus*. They performed in vitro and in vivo experiments using *Aedes albopictus* C6/36 cells, two strains of *Culex quinquefasciatus* (Sebring and Honduras), CxFV Izabal and West Nile virus isolate GU-06-2256. Replication of WNV was monitored between day 0 and 14 in CxFV-positive and CxFV-negative C6/36 cell and no significant inhibition of WNV growth was observed. In the in vivo experiments the two strains of *Culex quinquefasciatus* mosquitoes were intrathoracic inoculated with either CxFV, heat-inactivated CxFV or a mock infection, all groups were exposed to a WNV-infectious blood meal of multiple titres 7 days post inoculation (dpi). 2 weeks following WNV exposure, individual mosquito bodies, legs and saliva were measured for WNV growth by plaque titration on Vero cells representing infection, dissemination and transmission. Similarly, to the in vitro experiment, there were no difference in WNV growth or transmission, and they conclude that CxFV Izabal have no effect on vector competence for WNV in *Culex quinquefasciatus* [89]. However, in the paper they discuss the limitation of infecting the mosquitoes by intrathoracic inoculation and that naturally infected mosquitoes would be preferable due to earlier studies [90, 91] showing that the route of infection affect the outcome of arbovirus superinfection [89].

In regard to this, Bolling et al. performed a similar study, using a laboratory colony of *Culex pipiens* naturally infected with CxFV, to study transmission dynamics of CxFV as well as the effects of CxFV infection on vector competence of WNV. They first performed an in vitro experiment where CxFV infected C6/36 cells were challenged with WNV, the results showed that WNV growth curves in co-infected cells were significantly lower than WNV only infected cells between 84 and 156 h post infection, by 168 h post infection no difference could be observed. They followed up with an in vivo experiment using two *Culex pipiens* laboratory colonies, the CPCO (*Culex pipiens*-Colorado, CxFV-positive) and the CPIA (*Culex pipiens*-Iowa, CxFV-negative). Female mosquitoes from the two colonies were given a WNV infectious blood meal and infection, dissemination and transmission rates were compared at 7 and 14 dpi. Results after 7 dpi showed that dissemination rate was significantly lower in the CxFV-positive mosquitoes and the infection rate was also lower but not significantly. However, there was no difference in the estimated transmission rate, and after 14 dpi there was no statistically significant difference between the two colonies. Both the in vitro and in vivo experiments show an early time-point difference that may represent interference between CxFV and WNV, however, no difference in the transmission rates between CxFV-positive and CxFV-negative mosquitoes question the overall impact of CxFV on vector competence of WNV [65]. These two studies utilized the C6/36 cell lines in their in vitro experiments, this cell line is derived from *Aedes albopictus* mosquitoes, although WNV and CxFV are generally associated with *Culex* mosquitoes. Kuwata et al. studied the effect of CxFV in a *Culex* cell line derived from *Culex tritaeniorhynchus* embryos (NIID-CTR) [92]. They generated a NIID-CTR cell line persistently infected with CxFV, which showed no CPE and grew normally as compared to the original NIID-CTR. The CxFV-positive and the original cell lines were challenged with Japanese encephalitis virus (JEV) or DENV, cell growth and DENV/JEV replication were analysed every 24 h between day 1 and 7 dpi. Superinfection with JEV represented an arbovirus generally associated with *Culex* mosquitoes and DENV which is unrelated to *Culex* mosquitoes served as a control to compare the results of the JEV-CxFV system. Experimental results showed that CxFV-positive cells significantly enhanced replication and/or release of JEV at 4 dpi compared to the JEV-only infected cells. The increase of the JEV-titre appeared at the same time as a 25% decrease in cell number of the CxFV-positive cells, which may explain the massive increase by a release of JEV particles into the medium. The JEV-titre stayed significantly higher compared to the control throughout the experiment. CxFV-positive cells challenged with DENV also showed a significant higher titre after 4 dpi, however, cells showed no CPE and there was no reduction in cell number. From this they conclude that pre-existing CxFV infection do not suppress growth of a superinfecting flavivirus but rather enhance virus release from the NIID-CTR cells [93].

A study by Talavera et al. investigated the ability of Rift Valley fever virus (RVFV) to infect, disseminate and be transmitted by *Culex pipiens* pre-infected with CxFV. To evaluate if CxFV can interfere with a viruses from different genera, CxFV (Flavivirus) and RVFV (phlebovirus). Female *Culex pipiens* were intrathoracically infected with CxFV and then received a RVFV-infectious blood meal, 14 days post RVFV exposure the mosquitoes were dissected and analysed for infection, dissemination and transmission. Results showed no significant difference in any of the parameter analysed compared to the control and they conclude that CxFV have no effect on RVFV replication in *Culex pipiens* mosquitoes [94].

To draw any conclusion on the effect of CxFV is difficult, most studies are inconclusive or have conflicting result, e.g. Newman et al. saw a strong correlation between WNV and CxFV infection rates, where field caught mosquitoes in Chicago had a four-fold increased likelihood of CxFV infection of WNV positive mosquito pools, compared to WNV-negative pools [95]. While, Crockett et al. saw no association between WNV and CxFV infection rates in mosquitoes collected in south-eastern united states [96].

#### Palm Creek virus

Hobson-Peters et al. isolated an ISV from *Coquillettidia xanthogaster* mosquitoes in northern Australia and the virus was named Palm Creek virus (PCV) after its place of isolation. In the same paper, they showed that the use of PCV pre-infected C6/36 cells resulted in suppressed replication of both WNV and Murray Valley encephalitis virus (MVEV) by 10–43 folds compared to WNV or MVEV-only infected C6/36 cells [97].

Hall-Mendelin et al. did a follow up study and performed in vivo experiments studying vertical transmission, modes of transmission and WNV replication interference of PCV. They used relevant arbovirus vectors of Australia and focused on *Culex annulirostris*, which is an important vector of WNV and MVEV in that region. In the transmission experiments, no *Culex annulirostris* were infected by PCV when orally exposed via a PCV-infectious blood meal, and no progeny reared from PCV-infected *Culex annulirostris* were PCV positive, showing that PCV is probably host-specific to *Coquillettidia xanthogaster*. However, *Culex annulirostris* can support PCV infection and 100% were infected when intrathoracic inoculated. Interestingly, experimental results showed that PCV positive *Culex annulirostris* had a significantly lower WNV infection rate than PCV negative when given a WNV-infectious blood meal. To understand the PCV-mediated exclusion of WNV via the oral infection route they examined the tissue tropism of PCV with immunohistochemistry and found that the virus is specifically localized in the epithelial lining of the midgut and was not present in any other tissues [98]. The exact mechanism of PCV blocking of WNV is not revealed, we can, however, speculate that it is a competition of cellular resources between PCV and WNV [73], or that PCV upregulate the immune activation hindering establishment of WNV entry and replication [71, 72].

#### Nhumirim virus

Another ISV that has been evaluated for its capacity to supress replication of important arboviruses is Nhumirim virus (NHUV) that was isolated in the Pantanal region of Brazil [99, 100]. Kenney et al. preformed co-infection experiments in C6/36 cell with prior or concurrent infection of NHUV and challenged with either WNV, SLEV or JEV. NHUV showed to be a very potent inhibitor of all of these viruses with peak-titre differences translated to 1.5 million-fold reduction of WNV, 80-fold reduction of JEV and a 15,000-fold reduction for SLEV compared to the arbovirus only infected controls. A study done by Goenaga et al. further support the inhibition effect in vitro. Experiments, performed with C6/36 cells and the *Aedes albopictus* cell line C7/10, showed a 4000-fold decrease in WNV growth in both cell-lines pre-infected with NHUV. However, when *Culex pipiens* were co-inoculated intrathoracically with NHUV and WNV in a 10:1 ratio and processed at 14 dpi no significant difference in infection, dissemination and transmission was seen compared to the control. They further assessed the capacity of *Culex quinquefasciatus*, co-inoculated with NHUV and WNV, to become infected and transmit WNV. Mosquitoes were co-inoculated and harvested at 3, 5, 7 and 9 dpi, the results demonstrated 100% WNV infection at all time points, except at 3 dpi where a 91% infection rate was observed, this was significantly lower than the control. Although there was no significant difference in WNV titres in bodies and saliva they saw a trend of lower WNV titres in the saliva of co-infected mosquitoes and there was a significant difference in the transmission rate of mosquitoes that were co-infecting at 7 and 9 dpi compared to the control [101]. NHUV showed promising in vitro interference of superinfecting viruses, however, the in vivo experiment was not clear. Co-inoculated *Culex quinquefasciatus* showed a significantly lower transmission rate at the later time points compared to the control, which could indicate an interference of WNV replication in the salivary gland. This was however, not observed in the *Culex pipiens* mosquitoes, indicating that NHUV only have this effect in some *Culex* species. In the paper, they discuss that further studies with prior infection of NHUV instead of co-infection could increase the interfering effect and would also simulate a more natural infection [101].

#### Eilat virus

Eilat virus (EILV) is an insect-specific alphavirus that was isolated from a pool of *Anopheles coustani* mosquitoes caught in the Negev desert of Israel [28] and have been used as a backbone in a vaccine platform [102]. Nasar et al. investigated the ability of EILV to interfere with superinfecting alphaviruses such as Sindbis virus (SINV), chikungunya virus (CHIKV) and western (WEEV), eastern (EEEV) and Venezuelan equine encephalitis virus (VEEV) in C7/10 cells and in *Aedes aegypti* mosquitoes. To investigate heterologous interference, C7/10 cells were infected with EILV at a MOI of 10 or a mock infection, 24 h post the initial infection cells were superinfected with either SINV, VEEV, EEEV, WEEV or CHIKV at a MOI of 1 or 0.1. In vitro results showed that EILV infection reduced the superinfecting virus production by 10 to 10,000-fold and delayed replication kinetics at least 12–48 h regardless of virus or MOI. To investigate if similar results are achieved in vivo, *Aedes aegypti* mosquitoes were infected with EILV by intrathoracic inoculation and 7 days post inoculation mosquitoes were provided a blood meals containing CHIKV of 10^5^ PFU/ml. Infection rate and CHIKV dissemination were analysed and results showed that EILV infection delayed dissemination from the midgut by 3 days, however, after 5 and 7 days post superinfection the dissemination rates were higher or identical as compared to the mock infected group. In the paper, they discuss several interesting factors regarding why the EILV-mediated interference was not observed beyond 5 days post superinfection in mosquitoes. For example, that EILV replication may decrease over time which reduce the interfering capacity, or that EILV and CHIKV may have different cell tropisms and are therefore not competing for the cellular resources [31].

#### Dual ISV infection

Lastly, a study done by Schultz et al. evaluated the effect of dual insect-specific virus infection on replication of ZIKV, DENV-2 and La Crosse virus (LACV) in the *Aedes albopictus* derived cell line Aa23. Their cell-line was persistently infected with CFAV and was further inoculated with the Phasi Charoen like virus (PCLV) [103] generating a CFAV-PCLV positive Aa23 cell line. These and the Aa23 control cells (CFAV-only) were challenged with ZIKV, DENV and LACV at a low MOI of 0.1 and a high MOI of 10, respectively, and arbovirus growth was analysed after 3 and 6 dpi. They observed a 90% reduction of ZIKV growth at both the low and high MOI which was significantly lower than the control. DENV-2 had a similar reduction (90%) of growth at the low MOI, however, in the case of the high MOI no difference in growth compared to the control was observed. LACV had a complete inhibition at the low MOI and no virus growth was detectable in the CFAV-PCLV positive cells, the high MOI resulted in a 90-99.9% reduction of LACV growth. Dual-ISV infection of the two virus families *Flaviviridae* (CFAV) and a *Peribunyaviradae* (PCLV) showed a robust interference and in one case a complete blockage of arboviruses within the same genus [104].

The majority of all vector competence studies, done so far have used the *Aedes albopictus* C6/36 cell line (Table 1), this cell line has a defective RNAi response [105] which question the biological relevance from these experiments, due to the central role the RNAi plays in controlling arbovirus infection in the vector. The C6/36 cell line is very useful and easy to work with, but additional immune-competent mosquito cell lines such as the Aa23 [106], Aag2 [107] or HSU [108] should be included in studies regarding vector competence. Further, recent studies have showed that laboratory mosquito cell lines can be persistently infected with ISVs, without any CPE or affecting the cell growth. Schultz et al. screened their mosquito cell lines and discovered a persistent infection of CFAV and PCLV [104]. Further, Weger-Lucarelli et al. screened the four widely used mosquito cell lines C6/36, U4.4, Aag2 and HSU and confirmed that all four cell lines were persistently infected with several viruses [109]. This should to be considered when designing an experiment or analysing data generated from these cell lines.

Novel biocontrol strategies should aim to be inexpensive, effective, environmentally friendly, safe and self-sustaining. The use of ISVs as a tool for control of arbovirus transmission have the potential to fulfil these requirements, however, ISV research is still in an early stage and further research is needed before ISVs are implemented in real life settings.

## Concluding remarks

Today’s technologies have enabled a new era of viral discoveries, where large metagenomics studies of environmental samples or animals are both possible and affordable. Insect-specific viruses are a relative new group of viruses with many interesting properties that have the potential to be utilized to further understand the evolution of viruses and to possibly aid the prevention of arbovirus transmission.
